# Polyglucosan body myopathy 1 may cause cognitive impairment: a case report from China

**DOI:** 10.1186/s12891-020-03884-0

**Published:** 2021-01-07

**Authors:** Lin Chen, Nan Wang, Wenbin Hu, Xuen Yu, Renming Yang, Yongzhu Han, Yan Yan, Na Nian, Congbo Sha

**Affiliations:** 1 Department of Neurology, The Affiliated Hospital of the Neurology Institute of Anhui University of Chinese Medicine, 357 Changjiang Road, Hefei, Anhui P.R. China; 2 Department of Endocrinology, The First Affiliated Hospital of Anhui Medical University, 218 Jixi Road, Hefei, Anhui P.R. China

**Keywords:** Polyglucosan body myopathy, RBCK1, HOIL-1, Ubiquitin ligase

## Abstract

**Background:**

Polyglucosan body myopathy 1 (PGBM1) is a type of glycogen storage disease that can cause skeletal muscle myopathy and cardiomyopathy with or without immunodeficiency due to a pathogenic mutation in the RBCK1 gene. PGBM1 has been reported in only 14 European and American families, and no cognitive impairment phenotype was reported. Its prevalence in Asia is unknown.

**Case presentation:**

We report a Chinese boy with teenage onset of skeletal muscle myopathy and mild cognitive impairment. Whole-exome sequencing analysis identified a homozygous missense mutation in RBCK1 (c.1411G > A:p.Glu471Lys). A muscle biopsy indicated the accumulation of periodic acid-Schiff-positive material, which could be ubiquitinated by immunohistochemistry with an anti-ubiquitin antibody. In skeletal muscle tissue, HOIL-1 and HOIP protein levels were lower than those in the control, confirming the phenotype of an RBCK1 mutation. MRI revealed abnormal cerebral white matter signals. Immune system and cardiac examination found no abnormalities. The patient was diagnosed with PGBM1 with no effective treatment.

**Conclusions:**

This case from China with a novel homozygous missense mutation in RBCK1 extends the phenotypic spectrum and geographical distribution of PGBM 1, which may cause cerebral white matter changes and cognitive impairment.

## Background

Polyglucosan body myopathy 1 (PGBM1, OMIM: 615,895) is a rarely inherited myopathy presenting with skeletal myopathy and cardiomyopathy with or without an immune disorder. It is characterized by the accumulation of polyglucosan in the tissue [1, 2]. Polyglucosan is less branched than normal glycogen and may aggregate into polyglucosan bodies, which cannot be digested by alpha-amylase [3]. Polyglucosan body disease (PBD) is a type of glycogen storage disease (GSD) and is mainly caused by mutations in eight different human genes, namely, GYG1, GBE1, RBCK1, PFKM, EPM2A, EPM2B (NHLRC1), PRDM8, and PRKAG2 [1]. The RBCK1 gene, encoding the E3 ubiquitin ligase on chromosome 20p13, causes PGBM1 following a homozygous or compound heterozygous mutation [2, 4]. E3 ubiquitin ligase plays a key role in specific protein substrate recognition of ubiquitination modification, which is one of the most important posttranslational modifications of proteins [5].

Previous studies showed that skeletal muscle weakness and cardiomyopathy with or without autoinflammation and immunodeficiency occurred in 18 PGBM1 patients from 14 European and American families [2, 4, 6, 7]. To date, no study has reported that PGBM1 may cause cognitive impairment.

We describe a case of PGBM1 caused by a novel RBCK1 gene homozygous missense mutation presenting as skeletal myopathy with cerebral white matter changes and cognitive impairment that may be associated with the disease.

## Case presentation

A boy developed weakness in his lower limbs from 2015, at age 13, which gradually worsened. By the summer of 2018, he had difficulty getting up from a squatting position and climbing stairs; he had weakness in his upper limbs and atrophy of muscles in his limbs, which was more noticeable in his lower extremities. He could still walk alone and carry out activities in his daily life independently, but with a clear waddling gait for unlimited distances, until he was admitted to the hospital in November 2018. Since the onset of weakness, his academic performance has gradually declined. His grades in primary school were approximately the same as those of the majority of his peers, while after onset in 2015, his grades were worse than his peers in the same grade. In particular, his computing ability had declined significantly; for example, he could not count backward from 100 by sevens, in the Mini-Mental State Examination (MMSE).

On physical examination in November 2018, he showed symmetrical predominant hip-girdle weakness [Medical Research Council score of 4/5 in deltoid, biceps and triceps, 4+/5 in wrist extension and flexion, 4/5 in psoas, 3/5 in hip adductors and abductors, and quadriceps, and 4/5 in tibialis anterior, plantar flexor and extensor bilaterally] and muscular atrophy. He could not rise from the floor and had a positive Gowers sign. He did not have gastrocnemius hypertrophy, dysphonia, or dysphagia. His parents and older sister did not display symptoms of muscle weakness and atrophy.

His serum creatine kinase, pyruvate, lactic acid, alanine transferase, aspartate aminotransferase, and thyroid hormone levels were normal. The lymphocyte classification and count and immunoglobulin in the blood were normal. The needle electromyography recorded positive sharp waves and fibrillation potentials in the left vastus medialis at rest. The average duration of motor unit action potentials (MUAPs) of the biceps, deltoid and tibialis anterior muscle at small force contraction were shortened, and their amplitudes were normal, which indicated mild myopathy. The motor and sensory nerve conduction velocity and amplitude of the peripheral nerve were normal. His electrocardiogram and Doppler ultrasounds of the heart, liver and thyroid were normal. Both the Montreal Cognitive Assessment and the MMSE scores were 24 (the patient’s educational level was junior high school).

**Fig. 1:**
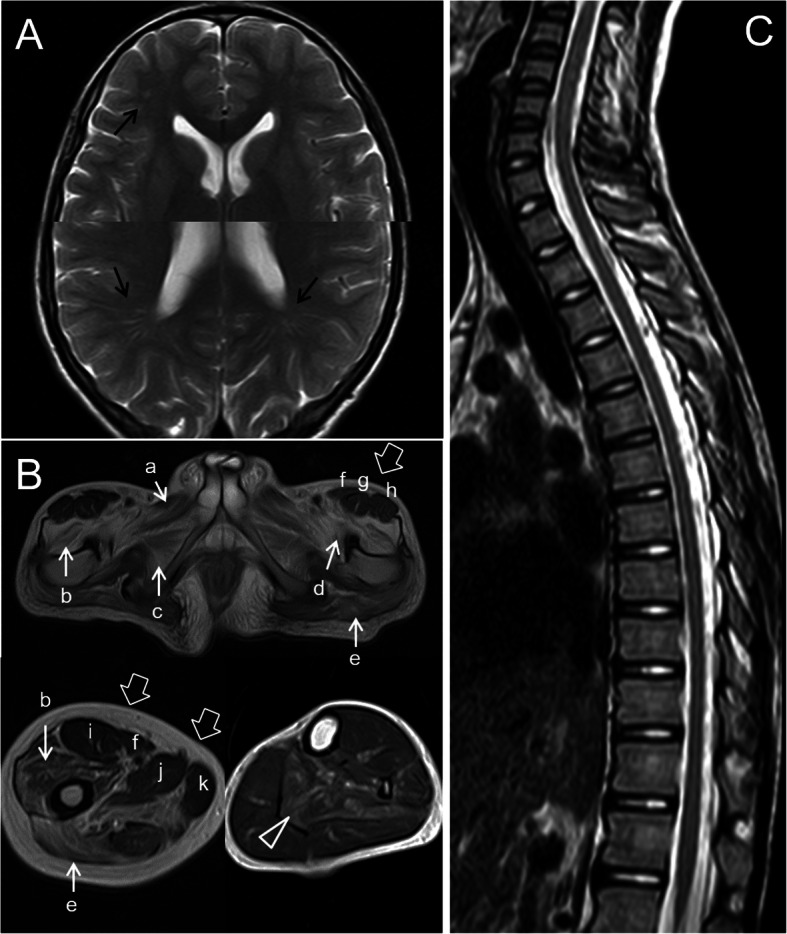
MRI of the brain, lower limb muscles and spinal cord. **a** Cerebral MRI showed abnormal signals in the subcortical white matter near the horns of the right lateral ventricle, which is high in T2-weighted imaging (black arrows), low in T1-weighted imaging and high in fluid attenuated inversion recovery (not shown). **b** The pelvic girdle and thigh muscles demonstrated obvious atrophy and fat infiltration of pectineus (**a**), vastus lateralis (**b**), iliopsoas (**c**), obturator externus (**d**) and gluteus maximus (**e**) (white arrows), while the sartorius (**f**), rectus femoris (**g, i**), tensor fascia lata (**h**), adductor longus (**j**) and gracilis (**k**) (white hollow arrows) were intact. Image of the legs revealed a moderate fat infiltration of the tibialis posterior (white hollow triangle). **c** The spinal cord did not exhibit atrophy

Brain MRI revealed punctate abnormal signals in the subcortical white matter of the horns of the lateral ventricle (Fig. 1a). The proximal muscles of the lower extremities and the pelvic girdle muscles showed marked atrophy and fat infiltration on MRI, while the calf muscle damage was lighter, and only the tibialis posterior represented moderate fat infiltration (Fig. 1b). The spinal cord had no atrophy (Fig. 1c).

**Fig. 2:**
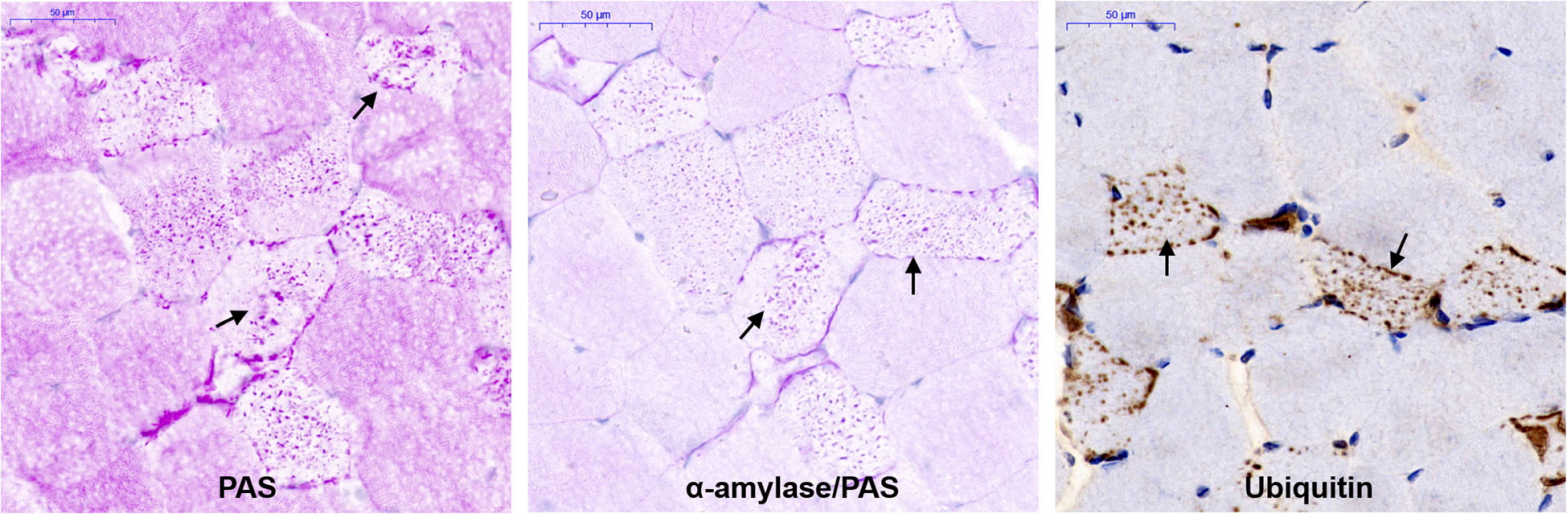
Morphological characteristics of skeletal muscle. **a** Staining of cryostat sections of the biceps with periodic acid-Schiff (PAS) shows that aberrant PAS-positive material (arrow) accumulates in numerous fibres. **b** Aberrant storage material is resistant to treatment with amylase (arrows). **c** The accumulated material can be ubiquitinated by immunohistochemistry with an anti-ubiquitin antibody (arrows)

This patient underwent open biopsy of the left biceps muscle. The morphology of the skeletal muscle was investigated by light microscopy. In skeletal muscle, there is some abnormal accumulation of periodic acid-Schiff (PAS)-positive material (Fig. 2a), which cannot be digested by alpha-amylase (Fig. 2b) and could be ubiquitinated by immunohistochemistry staining with an anti-ubiquitin antibody (Proteintech, #10201-2-AP) (Fig. 2c).

**Fig. 3:**
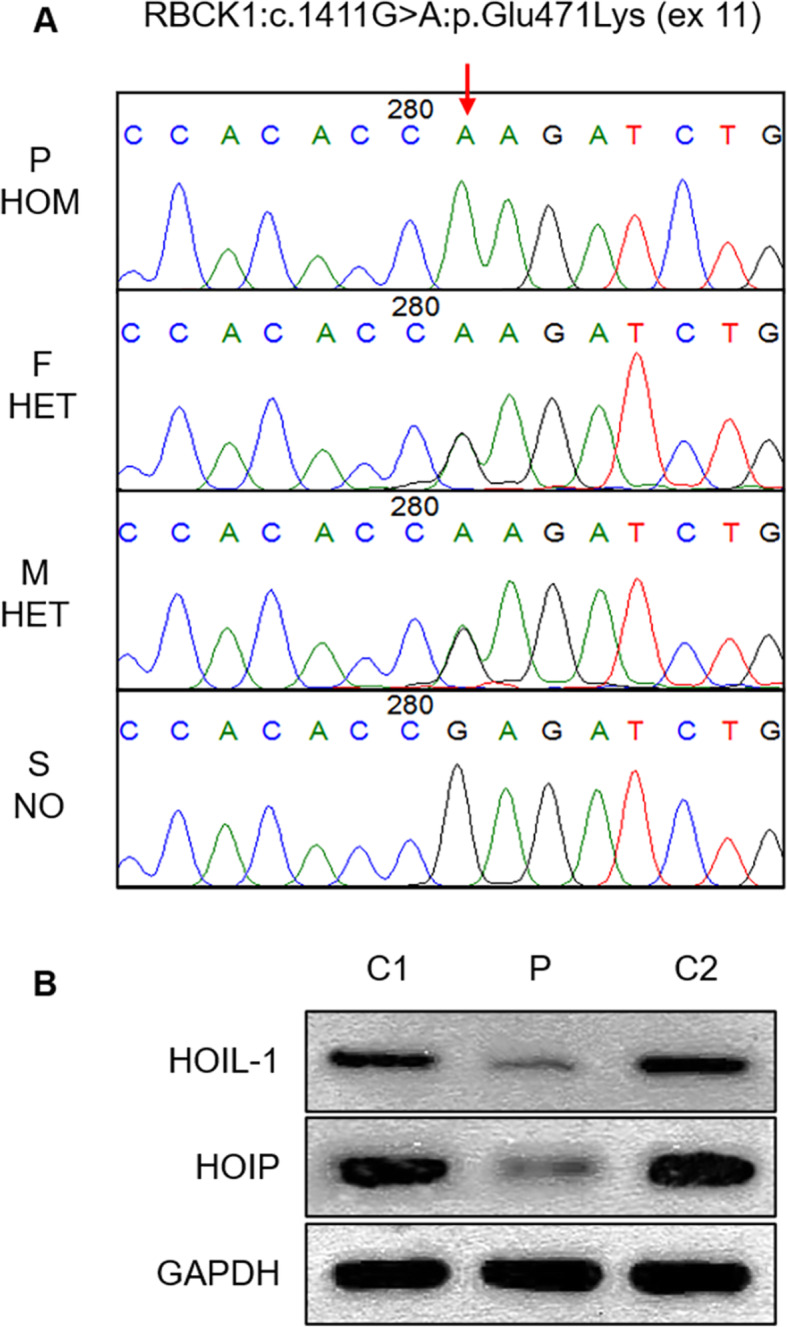
RBCK1 gene mutations and decreased expression of HOIL-1 and HOIP proteins. **a** RBCK1 gene homozygous missense mutation was found in the patient. The verification of related gene mutations in the family is shown. Red arrows represent mutation positions. P, patient; F, father; M, mother; S, sister; HOM, homozygous; HET, heterozygous; N, no mutation. **b** Low levels of HOIL-1 and HOIP protein expression in skeletal muscle tissues from patients (P) and controls (C1 and C2)

Whole-exome sequencing of this patient showed that he harboured a homozygous missense mutation in RBCK1 (NM_031229.3:c.1411G > A:p.Glu471Lys), which was submitted to ClinVar of National Center for Biotechnology Information (Accession: VCV000804223.1). His parents’ RBCK1 gene was heterozygous, and his sister had no mutation, which was verified by Sanger sequencing at the corresponding sites (Fig. 3a). In skeletal muscle tissue, HOIL-1 and HOIP protein levels were approximately 50% lower than those in two healthy control tissues detected by Western blot (WB). The WB images were turned into 8-bit images, and the grey value within each area was then measured. The patient’s grey values of HOIL-1 and HOIP in WB were 18,035 and 24,575, respectively, compared with 42,383 and 50,566 of control 1 and 52,186 and 47,427 of control 2 (HOIL-1 antibody, Proteintech, #26367-1-AP; HOIP antibody, Proteintech, #16289-1-AP) (Fig. 3b).

## Discussion and Conclusions

We identified a novel pathogenic homozygous mutation in RBCK1 from a Chinese PGBM1 case that may cause cerebral white matter changes and cognitive impairment.

This mutation has not been reported previously. A variant effect prediction analysis revealed probable damage (0.919/1) by PolyPhen2 and deleterious (-3.777, cut-off = -2.5) by SIFT. The diagnosis of PGBM1 can be confirmed by the clinical phenotype, pathology, autosomal recessive genetic model of the RBCK1 gene and the decreased expression of HOIL-1 and HOIP proteins.

The RBCK1 gene encodes heme-oxidized IRP2 ubiquitin ligase 1 (HOIL-1) and is related to HOIL-1L interacting protein (HOIP), which constitutes the linear ubiquitination chain assembly complex (LUBAC), a component of the NF-κB cascade involved in IKK complex activation [8–11]. NF-κB plays an important role in the regulation of the immune system [12]. Therefore, pathological mutations in RBCK1 can cause autoinflammation, immunodeficiency, and amylopectinosis [1, 4, 13], except for skeletal and cardiac muscle presentations.

Some studies have discovered that clinical phenotypic diversity may be partly correlated with the nature and location of RBCK1 mutations. N-terminal mutations of RBCK1 mainly cause immunological dysfunction; variants in the middle- or C-terminal regions may be prone to cardiomyopathy and neuromuscular symptoms. Additionally, truncating variants may generally result in more severe phenotypes than missense mutations, similar to most hereditary diseases [2, 6]. This patient’s mutation is closer to the C-terminus of the RBCK1 gene, similar to other patients, and he presented with neuromuscular symptoms, no immunological dysfunction and cardiomyopathy. We directly measured the RBCK1 gene expression products, HOIL1 and HOIP, in this patient’s skeletal muscle tissue by WB. His residual HOIL1 and HOIP proteins were approximately 50% of healthy controls’ (Fig. 3B), which are significantly better than the complete deficiency reported by Boisson in three patients with truncating variants near the N-terminal mutations that manifested as severe infections, systemic autoinflammation and myopathy and died during childhood [4]. This may be the reason why this patient only showed slowly progressive myopathy without cardiomyopathy and immunodeficiency.

He appeared to be affected by mild cognitive impairment and cerebral white matter changes (Fig. 1A). In PBD patients, leukoencephalopathy, spinal cord atrophy indicated by MRI, and mild cognitive impairment are common [14, 15], especially in adult polyglucosan body disease (APBD), but no previous study has reported these conditions in PGBM1 patients. This Chinese PGBM1 patient had minor cerebral white matter changes and mild cognitive impairment and no spinal cord atrophy. Unfortunately, we did not have sufficient evidence to prove that the pathogenic mutation in RBCK1 can cause cerebral white matter lesions and cognitive impairment. There was only a significant correlation between cognitive damage and the onset of myopathy in the timeline. In addition, MRI revealed cerebral white matter lesions, which are usually a cause of cognitive impairment and a common manifestation in PBD[15] and could not be explained by other causes. Further research on the association between PGBM1 and cognitive impairment will be considered in the future. It is still unclear whether the degree of leukoencephalopathy and cognitive impairment are related to the nature and location of the RBCK1 mutation.

In summary, this was a new case of PGBM1 caused by a novel mutation in RBCK1 from China, with associated cognitive impairment and cerebral white matter changes. At this stage, there seems to be insufficient evidence to conclusively link this mutation with the cerebral changes noted, and it may be that these changes relate to a different disease process.

## Data Availability

The datasets used and analysed during the current study are available from the corresponding author on reasonable request.

## Abbreviations

GSD: Glycogen storage disease
HOIL: Heme-oxidized IRP2 ubiquitin ligase
HOIP: HOIL-1L interacting protein
IKK: Inhibitor of kappa B kinase;
LUBAC: Linear ubiquitination chain assembly complex
MMSE: Mini-mental state examination
MRI: Magnetic resonance imaging
NF: Necrosis factor
PAS: Periodic acid-Schiff
PGBM: Polyglucosan body myopathy
PGD: Polyglucosan body disease
